# Malignant Pleural Mesothelioma: A Comprehensive Review

**DOI:** 10.3390/jcm13195837

**Published:** 2024-09-30

**Authors:** Molly Jain, Morgan Kay Crites, Patricia Rich, Bharat Bajantri

**Affiliations:** Parkview Health, Fort Wayne, IN 46845, USApatricia.rich@parkview.com (P.R.);

**Keywords:** malignant pleural mesothelioma, mesothelioma, asbestos, biomarkers, chemotherapy, surgery, radiotherapy

## Abstract

Mesotheliomas are hyperplastic tumors that envelop the serosal membranes that safeguard the body’s external surfaces. Although certain instances may exhibit indolent characteristics, a significant number of tumors demonstrate rapid progression and a poor prognosis. Mesotheliomas are typically categorized as benign or malignant, with malignant mesothelioma being more frequently linked to asbestos exposure. Malignant pleural mesothelioma (MPM) predominantly impacts males and often emerges in the late 50 s or beyond, characterized by a median age of early 70 s among patients exposed to asbestos lasting from 2 to 4 decades. Respiratory exposure to asbestos particles leads to the development of malignant mesothelioma, characterized by recurrent inflammation, disruption of cell division, activation of proto-oncogenes, and generation of free radicals. In pleural mesothelioma, BAP1, CDKN2A, and NF are the most often mutated genes. Accurate diagnosis and assessment usually require the use of chest computed tomography (CT) scans, magnetic resonance imaging (MRI), and positron emission tomography (PET). Radiation therapy, immunotherapy, chemotherapy, and surgery are some of the treatment options that are currently available. This systematic review provides a comprehensive analysis of the latest research, biomarkers, evaluation, and management strategies for malignant pleural mesothelioma.

## 1. Introduction

Mesotheliomas are proliferative neoplasms with variable outcomes depending on their specific type [1]. Although some cases could be slow-growing, many mesotheliomas have aggressive development patterns, which can lead to rapid deterioration in patient health. Mesotheliomas are comprised of epithelial and mesenchymal cells that cover the serosa that protect the body’s surfaces [1]. Mesotheliomas are classified as either benign or malignant [1]. Among the several benign mesotheliomas, benign multicystic peritoneal mesotheliomas (BMMPs) are the most prevalent [2]. Malignant mesothelioma (MM) is more typically related to asbestos exposure than benign mesothelioma, which has a variety of causes [2]. Males are the primary demographic affected by malignant pleural mesothelioma (MPM) [1]. MPM typically manifests in individuals in their fifties or later, with a median age of 72 and a history of exposure spanning 2 to 4 decades [1]. Women between the ages of 20 and 40 are more likely to be diagnosed with BMMP, which has been connected to endometriosis and pelvic inflammatory diseases [2]. Benign mesotheliomas affect older men, averaging 67 years of age [2].

The symptoms of mesothelioma may not manifest until 10–50 years after asbestos exposure, due to the extended incubation period associated with this type of disease [1,3]. MPM often manifests with chest discomfort and symptoms of respiratory distress [3]. One of the first symptoms that patients often experience is dyspnea, which can be a sign of pleural effusion, present in approximately 90% of patients [3]. Furthermore, non-specific symptoms include unintentional weight loss, decreased appetite, persistent cough, and exhaustion; the appearance of a lump on the chest wall may also be experienced by patients [1].

The inhalation of asbestos is a contributing factor to MPM, which results in the generation of free radicals, the activation of proto-oncogenes, the disruption of cell division, and recurrent inflammation of the pleura [1]. The diagnostic procedure involves a thorax CT scan using intravenous contrast, a thoracoscopic pleural biopsy, and, if necessary, thoracentesis of pleural effusion followed by cytologic laboratory analysis [1,2]. A chest CT scan will detect localized regions of thickness in the pleura, as well as a sizable invasive mass that is suggestive of an advanced-stage disease [1,3]. While MRI and laparoscopy are used to examine diaphragmatic invasion, PET scans are used for screening for metastatic disease [1]. A blood biomarker called megakaryocyte potentiating factor (MPF) is used to identify MPM [3].

Unfortunately, malignant mesothelioma has a grim prognosis, as it is highly fatal due to its aggressive activity and the fact that it is frequently detected at an advanced stage Mesothelioma has an extremely low five-year survival rate, approximately 10% [4]. Although there have been improvements in treatment options, the difficulties presented by tumor heterogeneity, limited effectiveness of therapies, and resistance mechanisms highlight the necessity for additional research and creative approaches, to enhance patient outcomes.

## 2. Materials and Methods

We conducted a keyword search on PUBMED using the following query: (“Mesothelioma”) OR (“Malignant pleural mesothelioma” OR “Mesothelioma, biomarkers/genomic analysis” OR “Mesothelioma, Malignant/radiotherapy” OR “Mesothelioma, Malignant/surgery” OR “Mesothelioma, Malignant/chemotherapy”).

Articles that were not written in English were excluded. Two authors (MJ, MC) subsequently selected all articles that were retrieved, based on the title and abstract. The final selection was made after reading the full text.

## 3. Pathophysiology

### 3.1. Asbestos and Mesothelioma

The six fibrous mineral types that make up asbestos include Tremolite (blue asbestos), Actinolite (brown asbestos), Anthophyllite, Crocidolite, and Chrysotile (white asbestos) [3]. There is a well-established link between exposure to amphibole and the emergence of MPM. The most potent carcinogen has been shown to be crocidolite [3,5]. Thinner and longer fibers, especially those longer than 8.0 μm and broader than 0.25 μm, are thought to be more harmful [3]. This is because they remain in the pleura for a longer period and can penetrate the lungs, leading to repeated tissue injury and repair, resulting in local inflammation.

### 3.2. Postulated Mechanism of Pathogenesis

The particular processes by which inflammation influences the course of MPM are not entirely known. Nonetheless, evidence supports a link between localized and systemic inflammatory responses and patient prognosis. Prolonged exposure to asbestos causes many biological changes in mesothelial cells, such as DNA damage, suppression of the cell cycle, and apoptosis [5]. Additionally, the body generates inflammatory mediators as a reaction to asbestos exposure [3,5]. An intense and persistent systemic inflammatory response, characterized by the migration of white blood cells and the secretion of cytokines, triggers the formation of malignant alterations in mesothelial cells [5]. Tumor-associated macrophages (TAMs), regulatory lymphocytes (Treg), and myeloid-derived suppressor cells (MDSCs) are recruited by malignant cells [5]. These cells aid in the formation of new blood vessels, extracellular matrix remodeling, immune system evasion, and finally tumor development [3,5].

In response to asbestos, mast cells (MCs) release nuclear factor-kB (NF-kB) and tumor necrosis factor-alpha (TNFA) [5]. Crocidolite induces localization of macrophages in the pleura and lung, leading to the subsequent production of TNFA [5]. Moreover, it stimulates mast cells to produce the TNFA receptor, TNF-R1, and to release TNFA, resulting in subsequent paracrine and autocrine reactions [5]. TNFA triggers the NF-kB pathway, allowing mast cells with DNA damage caused by asbestos to gradually develop into malignant mesothelioma [5]. By initiating the release of reactive oxygen species (ROS) and reactive nitrogen species (RNS), iron-catalyzed asbestos fibers can induce genotoxicity [3,5]. This can result in a wide range of mutations. As a result, it is suggested that the propensity of asbestos fibers to persist in the pleura for long periods of time causes their pathogenic character and results in repeating cycles of injury and healing at the site of inflammation [3,5].

### 3.3. Genomic Analysis in Malignant Mesothelioma

No particular oncogene has been found in MPM thus far, suggesting that MPM is a type of cancer resulting from the suppression of tumor suppressor genes instead than oncogenes activation. The genes most often mutated in MPM are BRCA-1-associated protein 1 (BAP1), Cyclin-Dependent Kinase inhibitor 2A (CDKN2A), and Neurofibromatosis type 2 (NF2) [6].

BAP1 is a member of the c-terminal hydrolase family present in DNA repair complexes linked to BRCA1. It acts as a deubiquitinase enzyme [3]. The association between BAP1 and reduced tumor growth has been established in several experimental models, and it engages with proteins involved in the regulation of the cell cycle. BAP1 will influence a wide variety of cellular functions, such as DNA repair, cell cycle progression, chromatin remodeling, and cell differentiation [6]. The pivotal function of the BAP1 protein as an inhibitor of apoptosis triggered by metabolic stress is well recognized [6]. There is a highly penetrant MPM syndrome linked to germline mutations in BAP1 [6]. Three different research groups looking at MPM, cutaneous melanoma, and uveal melanoma discovered the BAP1 tumor predisposition syndrome (BAP1-TPDS), MPM, cutaneous melanoma, and uveal melanoma [3,6].

Evidence of Programmed Death-Ligand 1 (PD-L1) overexpression has been detected in MPM, along with several additional mutations such as Ewing Sarcoma breakpoint region 1-Activating Transcription Factor 1 (EWSR1-ATF1) and FUS RNA binding protein (FUS) and ATF1 (FUS-ATF1) fusions [3]. CDKN2A loss is observed in MPM; however, it is less prevalent than in pleural mesothelioma cases [3].

## 4. Diagnosis and Evaluation

### 4.1. Clinical Presentation

Most individuals experience a non-specific dry cough [1]. Shortness of breath is also prevalent. Chest wall discomfort is a distinct symptom typically characterized as localized soreness [1,3,5]. An underlying pleural effusion can cause palpable soft tissue fullness or bulk, as well as reduced breathing sounds on auscultation and dullness to percussion [1,4]. Some patients experience the development of splinting and even scoliosis towards the affected side of the body [1]. Pleural effusions are common and primarily manifest on the right side in 60% of instances [1]. A bilateral effusion may be seen in five percent of cases. Numerous paraneoplastic conditions, including hypoglycemia, autoimmune hemolytic anemia, hypercalcemia, hypercoagulable states, and disseminated intravascular coagulation, have been linked to mesothelioma [3,7].

### 4.2. Imaging

In the advanced phases of the disease, a chest CT scan will demonstrate a substantial invasive mass, as well as localized regions of pleural thickening [3,7]. Magnetic resonance imaging can accurately determine the extent of invasion of the diaphragm or mediastinal tissues, which is crucial for evaluation of patients being considered for surgery [7]. PET imaging is valuable, due to the hypermetabolic features of mesothelioma [7]. PET can be used for both staging and post-treatment monitoring [1,3,7].

During the initial work-up, it is recommended to perform ultrasound-guided thoracentesis and examine the pleural effusion for cytopathological analysis [3,7]. Nevertheless, it is crucial to acknowledge that this method has a low sensitivity and is capable of accurately diagnosing just under a third of MPM cases. Although it is not extremely accurate for diagnosis draining, the pleural effusion might alleviate symptoms such as chest tightness or difficulty breathing [3].

### 4.3. Histopathological Analysis

The epithelioid histologic type is the most frequently observed and is linked to the most favorable outcome. Sarcomatoid variations displaying distinctive spindle shape tend to have a more unfavorable prognosis. Frequently, a combination of epithelioid and sarcomatoid histologies might be observed [3,7].

Epithelioid mesotheliomas have unique features in their structure, cellular composition, and neighboring tissue, which might cause ambiguity when distinguishing them from other tumor types [3]. Sarcomatoid mesotheliomas are characterized by elongated cells that are arranged in bundles or in a chaotic pattern [7]. These cells exhibit varying degrees of abnormality in their appearance, ranging from mild to severe, and may also contain different types of tissue. Biphasic mesothelioma requires at least 10% of both sarcomatoid and epithelial components [3]. However, desmoplastic mesothelioma must contain a minimum of 50% hyalinized fibrous stroma. Patients with sarcomatoid and biphasic tumors have much poorer survival rates than those with epithelioid mesothelioma [8].

Determining the histopathological diagnosis of mesothelial lesions is challenging, especially when differentiating between malignant lesions and benign tumors, as well as reactive mesothelial hyperplasia or reactive fibrous pleurisy [3]. The differentiation between reactive hyperplastic mesothelium and mesothelioma in pleural biopsies can be challenging, due to the presence of cytologic atypia, enhanced cellularity, and mitosis in both diseases [3]. Essential criteria that might not always be available in biopsies are evaluating infiltration features, vascular pattern, growth pattern, degree of necrosis, and papillae characteristics [3].

### 4.4. Cytology and Immunohistochemistry

Immunohistochemistry is necessary for diagnosing complex malignant mesothelioma cases that involve various types of malignancies, including carcinomas, sarcomas, melanomas, and lymphomas, both of epithelial and nonepithelial type [9]. Diagnosing MPM requires multiple IHC markers. One marker alone is not accurate enough. Therefore, it is recommended to use panels consisting of at least two carcinoma markers (such as pCEA BER-EP4, MOC-31, Claudin 4, HEG1) and two mesothelial markers (such as WT1, calretinin, CK5/6, D2-40) [3].

Mesotheliomas can exhibit the GATA-3 antibody, which is also present in breast and urothelial carcinomas. A single pleural tumor may have characteristics that are similar to those of sarcomatoid mesothelioma. However, it possesses NAB2-STAT6 gene fusion and exhibits positive staining for STAT6 and CD34 [3]. The molecular testing for SYT-SSX1 or SYT-SSX2 fusions can be used to differentiate between sarcomatoid and biphasic mesothelioma and synovial sarcoma (monophasic and biphasic). The presence of TLE1 nuclear staining allows for the detection of these fusions [3,5].

### 4.5. Staging

In order to complete the clinical staging and establish the histology, medical procedures such as thoracoscopy, open pleural biopsy, mediastinal staging using bronchoscopy, and/or fine needle biopsy may be conducted. The International Mesothelioma Interest Group (IMIG) and the International Association for the Study of Lung Cancer (IASLC) jointly created the current and extensively used TNM staging system in 1994. It was amended in Version 1.2024 in 2023 [3,7].

- Stage IA is characterized by a tumor that is exclusive to the ipsilateral parietal pleura, and may or may not affect the visceral, mediastinal, or diaphragmatic pleura. No distant metastasis (M0) or lymph node metastases (N0) have been identified.
- Stage IB describes a tumor affecting all the ipsilateral pleural surfaces (parietal, mediastinal, diaphragmatic, and visceral pleural), with at least one of the designated features: diaphragmatic muscle involvement or tumor migration from the visceral pleural layer into the underlying pulmonary parenchyma. No lymph node metastases (N0) or distant metastasis (M0) is detected.
- Stage II has all the characteristics of a Stage I tumor, but also includes the spread of cancer cells in ipsilateral bronchopulmonary, hilar, or mediastinal lymph nodes (N1). No distant metastases are present (M0).
- Stage IIIA refers to a tumor that has spread extensively, involving all ipsilateral pleural surfaces. It may also involve the endothoracic fascia, extend into the fat surrounding the mediastinum, have a single area of tumor that can be resectable and which extends into the chest wall, or have nontransmural involvement of the pericardium. There is no distant metastasis (M0), but the malignancy has metastasized to ipsilateral bronchopulmonary, hilar, or mediastinal lymph nodes (N1).
- Stage IIIB denotes a malignancy with comparable local advance to that of Stage IIIA, but with metastases in the contralateral or ipsilateral/contralateral supraclavicular lymph nodes (N2). This stage has comparable local advancement to that of Stage IIIA. No distant metastasis is present (M0).
- Stage IV signifies the presence of a highly advanced tumor that cannot be surgically removed, which has spread to all ipsilateral pleural surfaces. It also includes one or more severe extensions, such as widespread infiltration into the chest wall, destruction of ribs, direct spread to the contralateral pleura, mediastinal organs, spine, or pericardium. Distant metastasis (M1) is present.
- Surgical procedures for staging, including mediastinoscopy and laparoscopic peritoneal wash or lavage, are recommended to more accurately stage mesothelioma patients. These techniques help detect early microscopic cancer spread that might be missed through imaging or visual inspection during surgery. Identifying such metastases refines the staging process, improves the accuracy of prognosis, and aids in determining the most appropriate treatment strategies. This can be especially valuable in aggressive cases, potentially avoiding unnecessary and high-risk surgeries that would offer little benefit to patients [9,10].

### 4.6. Biomarkers in Research

Biomarkers serve as valuable diagnostic and prognostic instruments for various forms of cancer. Regrettably, various serum and pleural biomarkers have shown limited sensitivity and specificity in diagnosing MPM [11]. The most promising biomarker is mesothelin, a glycoprotein that is present on the cell surface of MPM [12]. The presence of mesothelin can be identified in both serum and pleural samples, in addition to fibulin-3 [13]. Megakaryocyte potentiating factor, a variant of the mesothelin precursor protein, and the glycoproteins osteopontin and fibulin 3 are additional blood-based biomarkers that have been investigated in MPM [12,13]. Fibulin 3 is also present in the pleural fluid; elevated levels of this protein are associated with more severe stages of the disease [12,13].

## 5. Treatment

### 5.1. Surgery

The presence of mediastinal lymph node involvement is a poor prognostic factor in MPM, so patients who meet the criteria for surgery should undergo either EBUS (endobronchial ultrasonography) or mediastinoscopy [3,12]. The three primary surgical techniques employed to treat resectable mesothelioma tumors are pleurectomy/decortication, thoracoscopy with pleurodesis, and extrapleural pneumonectomy [13,14].

The MARS 2 trial compared extended pleurectomy decortication (EPD) plus chemotherapy to chemotherapy alone, in treating pleural mesothelioma [15]. Woodard and colleagues conducted a review of the National Cancer Database (NCDB) for patients diagnosed with mesothelioma between 2010 and 2016, identifying 943 patients who underwent surgical management and 554 patients who were managed non-surgically, all of whom survived for at least five years. Their findings suggest that long-term survival is possible, particularly in patients with early-stage mesothelioma and favorable epithelial histology, regardless of whether they underwent surgery or not [16]. The results indicated that EPD in conjunction with chemotherapy led to inferior survival rates, an increased occurrence of severe adverse events, and reduced quality of life, relative to chemotherapy alone [13]. In particular, patients who underwent surgery had a median survival of 19.3 months (approximately 1.5 years), while those who received only chemotherapy had a median survival of 24.8 months (approximately 2 years) [15]. The study concluded that EPD should not be recommended for pleural mesothelioma patients, advocating for systemic treatments instead [13]. Proponents of surgery argue that it could be crucial for long-term survival, contrary to the conclusions drawn by the MARS 2 trial. However, there are not enough data to definitively state that surgical approaches should be excluded from treatment plans for malignant mesothelioma. One limitation of the MARS 2 trial is that it did not specify the type of surgical procedures used, whether pleurectomy/decortication (P/D) or extended pleurectomy/decortication (EP/D), nor did it clarify whether the surgeries were performed through formal thoracotomy or video-assisted thoracoscopic surgery (VATS) [17]. Studies, such as one by Halstead et al., have shown that VATS pleurectomy–decortication was associated with fewer post-operative complications and shorter hospital stays, with a median of 8 days, compared to the nearly 2-week stay seen in the MARS 2 trial [18]. Additionally, patients who underwent surgery often experienced symptoms like pain, fatigue, shortness of breath, insomnia, and appetite loss, though these were reportedly less common in those who had minimally invasive procedures. A significant 39% of the surgical group did not receive post-operative chemotherapy, which is typically recommended, likely due to the difficult recovery process. Moreover, fewer patients in the surgical group received immunotherapy. The trial also did not provide detailed information on the histological types of mesothelioma, particularly the aggressive sarcomatoid subtype, which is associated with a worse prognosis, regardless of treatment choice. The proportion of patients with sarcomatoid histology in the surgical group remains unclear [19].

Pleurectomy/decortication is a surgical operation performed with the goal of minimizing the amount of tumor present in the body [3,20]. This treatment entails an open thoracotomy and the removal of the parietal pleura. The procedure encompasses the area close to the mediastinum, pericardium, and diaphragm (perhaps necessitating partial diaphragm resection), together with the excision of the visceral pleura to decorticate the lung [3,15]. This treatment provides relief from localized symptoms and aids in the prevention of the recurrence of pleural effusion. Nevertheless, it is generally not regarded as curative, and it typically leads to a high incidence of local (80% to 90%) or distant (10% to 36%) recurrence [3].

Extrapleural pneumonectomy (EPP) is a procedure that entails a thorough excision of tissue in the hemithorax [3]. It includes the diseased lung, mediastinal lymph nodes, diaphragm, pericardium, and visceral and parietal pleura [3,21]. It is considered a more severe treatment for this purpose. Patients with comorbidities that limit their activities, low performance status, mediastinal lymph node involvement, or sarcomatoid histology are frequently restricted from this treatment because of an elevated risk of complications, including mortality, and a diminished prognosis [21].

Pleurodesis is a surgical intervention used to eliminate fluid accumulation in the pleural cavity [3]. The technique involves extraction of fluid by thoracoscopy, carried out under general anesthesia or sedation. Alternatively, a thoracic tube can be inserted through a thoracostomy to achieve the same outcome [3,21]. Following the removal of fluid from the pleural cavity, sclerosing drugs are introduced to prevent additional fluid collection [18].

### 5.2. Radiotherapy

The principal indications for radiotherapy in malignant pleural mesothelioma (MPM) encompass the following:

- Hemithoracic radiotherapy preceding or succeeding extrapleural pneumonectomy;
- Hemithoracic radiotherapy subsequent to decortication/pleurectomy;
- Palliative radiotherapy to mitigate local symptoms [3,22].

Extrapleural pneumonectomy can be combined with radical hemithoracic radiation (RHR) to improve local control [23]. This therapy is linked to in-field failure rates of 15% to 35% [3]. The justification for administering neoadjuvant hemithoracic radiation before extrapleural pneumonectomy stems from the observation of recurrent tumor metastases to the contralateral lung and peritoneum, potentially associated with the surgical intervention [3,23,24]. In light of the controversial results of the MARS-1 trial, the application of extrapleural pneumonectomy has diminished in recent years, favoring lung-sparing methods such as pleurectomy and lung decortication [3,25]. The IMPRINT study, a prospective phase II trial, confirmed the safety of concurrently administering intensity-modulated radiation treatment to half of the chest along with chemotherapy, in patients who had previously undergone pleurectomy and lung decortication [3,26].

Radiotherapy can be employed in the palliative setting to manage the many symptoms that may not be effectively treated with drugs [1]. These symptoms include chest pain caused by chest wall invasion, hemoptysis, cough, and dyspnea. It can also be used in the setting of spinal cord compression [1,3].

### 5.3. Chemotherapy/Immunotherapy

Chemotherapy can be used as a preoperative or postoperative treatment for patients diagnosed with mesothelioma. A 2020 analysis compared survival in patients deemed surgical candidates (Stages I–III) who had neoadjuvant chemotherapy, versus those who underwent routine resection. It found that overall survival was similar, but post-surgical survival was worse in patients who received neoadjuvant treatment [27]. It is believed that these outcomes are due to a lack of response to chemotherapy, meaning continued progression while waiting to complete surgery. During the time a patient receives neoadjuvant chemotherapy their symptoms may continue to worsen if the cancer is not responding to the treatment, causing worsening of overall outcomes [27,28]. There is a continued need for evaluation and further research to confirm these findings and provide better outcomes for patients.

Chemotherapy and immunotherapy remain the mainstay of treatment for unresectable mesothelioma. Both systemic treatments are considered category one recommendations for unresectable malignant mesothelioma, according to the National Comprehensive Cancer Network (NCCN) [29]. Platinum-based chemotherapeutic drugs such as cisplatin are used in conjunction with pemetrexed [29,30]. Carboplatin can serve as a replacement for cisplatin in older patients, patients with comorbidities, or those who encounter toxicity with cisplatin, as it is often more well-tolerated [29,30]. Malignant pleural mesothelioma demonstrates increased resistance to treatment, leading to uncertain improvement in survival rates [1,3]. For instance, when gemcitabine and cisplatin are used together, the response rate ranges from 12% to 48% [31]. However, the median survival remains limited to 9 to 13 months [3,31]. Research has been conducted to discover if there are medications that can be added to chemotherapy to help improve response rates.

Bevacizumab is a vascular endothelial growth factor (VEGF) inhibitor that can be used with chemotherapy for mesothelioma treatment. VEGF is crucial in MPM, as it facilitates angiogenesis and stimulates the growth of tumors [31]. Bevacizumab is a type of monoclonal antibody that specifically targets VEGF (vascular endothelial growth factor). Recent studies have shown that it is effective in treating MPM (malignant pleural mesothelioma) [3,24,32,33]. The MAPS trial, a phase III multicenter study, randomly assigned 448 people with malignant pleural mesothelioma (MPM) to receive treatment with cisplatin and pemetrexed, either with or without bevacizumab [3,34]. Patients who received bevacizumab had a significantly longer median overall survival of 18.8 months (95% CI: 15.9–22.6) compared to 16.1 months (95% CI: 14.0–17.9) in the group that only received chemotherapy (*p* = 0.017) [3,34]. The progression-free survival (PFS) of 9.2 (8.5–10.5) months in patients who received bevacizumab alongside chemotherapy was significantly longer than that of 7.3 (6.7–8.0) months in those who received standard care (*p* < 0.0001) [3,34]. The incidence of adverse events was comparable between the groups; however, bevacizumab was associated with a higher prevalence of thromboembolic problems and kidney impairment [3,34]. The trials conclude that the use of bevacizumab is justified in combination with first-line conventional chemotherapy for patients with unresectable MPM [34].

Hyperthermic intraoperative chemotherapy (HITHOC) is a specialized treatment that combines several approaches to target cancer that has spread to the pleural space (the area around the lungs). This technique involves applying heated chemotherapy directly to the affected area during surgery, aiming to enhance the treatment’s effectiveness by using heat to increase the cancer-fighting power of the drugs. By combining chemotherapy with heat and, sometimes, surgical removal of tumors, HITHOC provides a promising option for improving symptoms and quality of life in patients who have limited treatment options. While this approach offers reasonable outcomes, particularly in reducing symptoms and improving survival rates, its full potential is still being researched. Ongoing studies are looking at how HITHOC could be enhanced with targeted therapies or immunotherapy, but more evidence is needed to elevate its recommendation as a standard treatment [35].

The systemic treatment options for unresectable MPM remain limited. Even with medications to counteract their side effects, many patients remain hesitant to start chemotherapy, which can limit their treatment options even further [36]. The CheckMate7433 trial led to the approval of double immunotherapy using nivolumab and ipilimumab in the treatment of unresectable mesothelioma [36,37]. Nivolumab and ipilimumab work to improve the function and activation of T cells in the immune system, prompting the body to fight cancer cells [37,38]. Immunotherapy does come with its own panel of side effects, but could be a better option for patients with reduced performance status or contraindications to pursuing chemotherapy treatment [39] (Table 1).

## 6. Conclusions

To summarize, mesothelioma, a cancer mostly associated with asbestos exposure, necessitates a comprehensive approach for its diagnosis and treatment. Precise and timely diagnosis is essential, facilitated by sophisticated imaging techniques such as PET-CT, MRI, and thoracoscopy for evaluating illnesses. Surgical procedures, such as cytoreductive surgery and pleurectomy/decortication, are performed to eliminate visible tumors and enhance the therapy of the disease in the affected region. Adjuvant therapies, such as chemotherapy and radiation, are specifically developed to target and treat any remaining microscopic disease.

Targeted medicines that disrupt specific signaling pathways demonstrate potential in many genetic subtypes. Although there has been significant advancement, mesothelioma continues to pose challenges because of its persistent nature, limited treatment choices, mechanisms of resistance, and delayed detection, leading to a bleak outlook. Finally, when individual patient needs are addressed, a multidisciplinary method will provide a stronger outcome and better quality of life.

## Figures and Tables

**Table 1:** Clinical trials related to Malignant Mesothelioma [40].

Status	Study Title	NCT Number	Sponsor	Conclusion
Completed	Cisplatin With or Without Pemetrexed Disodium	NCT00005636	Memorial Sloan Kettering Cancer Center	Demonstrated the efficacy of cisplatin and pemetrexed in managing malignant mesothelioma.
	NGR015: Study in Second Line for Advanced Malignant Pleural Mesothelioma	NCT01098266	AGC Biologics S.p.A.	Provided insights into the effectiveness of NGR-hTNF in second-line treatment for pretreated patients.
	Ph 2/3 Study on ADI-PEG 20 With Pemetrexed and Cisplatin	NCT02709512	Polaris Group	Suggested potential benefits of combining ADI-PEG 20 with standard chemotherapy in mesothelioma treatment.
	Nivolumab Combined With Ipilimumab vs. Pemetrexed and Cisplatin	NCT02899299	Bristol-Myers Squibb	Showed promise for immunotherapy combinations in enhancing treatment outcomes for unresectable mesothelioma.
	Early Palliative Care With Standard Care or Standard Care Alone in Improving Quality of Life of Patients with Incurable Lung or Non-colorectal Gastrointestinal Cancer and Their Family Caregivers	NCT02349412	Alliance for Clinical Trials in Oncology	Demonstrated that early palliative care significantly improves quality of life for patients and their caregivers.
	Fentanyl Sublingual Spray in Treating Patients With Breakthrough Cancer Pain	NCT00538850	INSYS Therapeutics Inc	Showed that fentanyl sublingual spray effectively manages breakthrough cancer pain, offering an alternative to traditional methods.
Active, Not Recruiting	DuRvalumab With Chemotherapy	NCT04334759	PrECOG, LLC	Investigating the role of immunotherapy alongside chemotherapy, which could lead to improved survival rates.
	Efficacy & Safety of rAd-IFN With Celecoxib & Gemcitabine	NCT03710876	Trizell Ltd.	Aiming to assess the combined impact of biological and chemotherapeutic agents on treatment efficacy.
Recruiting	MEDI5752 in Combination With Carboplatin Plus Pemetrexed	NCT06097728	AstraZeneca	Actively seeking participants to evaluate a new therapeutic combination that may enhance treatment effectiveness.
Terminated	Nintedanib (BIBF 1120) in Mesothelioma	NCT01907100	Boehringer Ingelheim	Termination highlights challenges in study design or recruitment, suggesting limitations in nintedanib’s application.
	Testing Addition of Targeted Radiation Therapy	NCT04158141	NRG Oncology	Termination reflects difficulties in integrating targeted radiation with standard treatments in early-stage mesothelioma.
Unknown	ONCONASE Plus Doxorubicin Versus Doxorubicin Alone	NCT00003034	Alfacell	The unclear status suggests potential issues with trial progression or reporting, warranting further investigation.

## Data Availability

No new data were created or analyzed in this study. Data sharing is not applicable to this article.
